# MioC and GidA proteins promote cell division in *E. coli*

**DOI:** 10.3389/fmicb.2015.00516

**Published:** 2015-05-28

**Authors:** Mark Lies, Bryan J. Visser, Mohan C. Joshi, David Magnan, David Bates

**Affiliations:** ^1^Molecular and Human Genetics, Baylor College of MedicineHouston, TX, USA; ^2^Integrative Molecular and Biomedical Sciences, Baylor College of MedicineHouston, TX, USA

**Keywords:** mioC, gidA, bacterial cell division, bacterial cell cycle, *oriC*, FIS, replication initiation, ymgF

## Abstract

The well-conserved genes surrounding the *E. coli* replication origin, *mioC* and *gidA*, do not normally affect chromosome replication and have little known function. We report that *mioC* and *gidA* mutants exhibit a moderate cell division inhibition phenotype. Cell elongation is exacerbated by a *fis* deletion, likely owing to delayed replication and subsequent cell cycle stress. Measurements of replication initiation frequency and origin segregation indicate that *mioC* and *gidA* do not inhibit cell division through any effect on *oriC* function. Division inhibition is also independent of the two known replication/cell division checkpoints, SOS and nucleoid occlusion. Complementation analysis indicates that *mioC* and *gidA* affect cell division *in trans*, indicating their effect is at the protein level. Transcriptome analysis by RNA sequencing showed that expression of a cell division septum component, YmgF, is significantly altered in *mioC* and *gidA* mutants. Our data reveal new roles for the gene products of *gidA* and *mioC* in the division apparatus, and we propose that their expression, cyclically regulated by chromatin remodeling at *oriC*, is part of a cell cycle regulatory program coordinating replication initiation and cell division.

## Introduction

In all cells, DNA replication and cell division are temporally coordinated to maintain a one-to-one relationship between genome and cell duplication. Cell cycle regulation in bacteria is subject to additional stringency because division cycles can be as short as 20 min, thus cells must replicate and segregate a full genome equivalent within this timeframe. To date no mechanism has been identified in *E. coli* to link replication and division. Pioneering work by the Helmstetter lab (Cooper and Helmstetter, 1968) indicated that the bacterial cell cycle might be controlled solely by the frequency and timing of replication initiation. This idea stemmed from synchronized cell experiments, which showed that in *E. coli* B/r strains the periods of DNA replication and septum development were relatively constant (~40 and 20 min, respectively) with the remainder of the cell cycle defined as a flexible pre-initiation “B” period (Dix and Helmstetter, 1973). It was hypothesized that cell division was triggered by an unknown event occurring at the end of the replication period, presumably replication of an essential cell division gene (Dix and Helmstetter, 1973; Den Blaauwen et al., 1999). Supporting this view, replication termination and cell division occur at the same cell location (Bates and Kleckner, 2005; Wang et al., 2005), and there is even some sharing of machinery between the two processes (e.g., FtsK translocase, Wang et al., 2005, 2006; Burton et al., 2007).

However, two lines of evidence suggest that cell division is initiated independently of replication termination. First, all known physical interactions between replicating DNA and the division apparatus are inhibitory. Specifically, midcell FtsZ polymerization is repressed by the presence of unsegregated DNA (Mulder and Woldringh, 1989; Moriya et al., 2010; Cambridge et al., 2014), partially dependent on direct inhibition from the nucleoid-bound SlmA protein in *E. coli* (Bernhardt and De Boer, 2005) or NOC in *B. subtilis* (Wu et al., 2009; Rodrigues and Harry, 2012). Second, most genetic and cytological data places initial FtsZ ring assembly steps very early in the replication period far in advance of termination (e.g., Addinall and Lutkenhaus, 1996; Yu et al., 1998; Harry et al., 1999). Inhibiting DNA replication prior to or soon after the initiation step imparts a strong cell division block independent of nucleoid occlusion, but once established, inhibition of replication elongation through drug treatment or temperature sensitive replisome mutant does not itself inhibit FtsZ ring assembly (Harry et al., 1999; Regamey et al., 2000; Arjes et al., 2014; Morigen et al., 2014). In such a cell, Z-rings form off-center in a nucleoid occlusion-dependent process, generating anucleate cells (Mulder and Woldringh, 1989).

**Figure 1:**
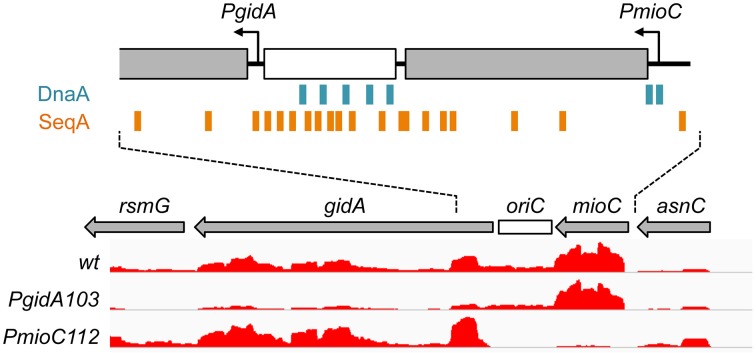
**Transcription in the** ***oriC*** **region**. The minimal *oriC* (white box) and surrounding genes are shown, with transcription direction indicated by arrows. Binding sites for DnaA (blue boxes) and SeqA (orange boxes) overlap *mioC* and *gidA* promoters, respectively. Relative transcription in *wildtype* and *gidA* and *mioC* promoter mutants, as determined by RNA-sequencing is shown (height of red lines indicate relative reads per base pair).

One possible connection between replication initiation and cell division is through activated expression of a cell division regulator gene near the replication origin, *oriC*. The very highly conserved *gidA* gene, which is located immediately leftward of *oriC* (Figure 1), was previously implicated in cell division via a cell filamentation phenotype in *gidA* (glucose inhibited division) deletion mutants when grown in glucose-containing media (Von Meyenburg and Hansen, 1980). The mechanism of the division defect in *gidA* mutants is unclear. Wild-type *gidA*, also known as *mnmG*, encodes a protein that in combination with MnmE is involved in modification of specific tRNA molecules, (Bregeon et al., 2001; Moukadiri et al., 2009). This tRNA modification may be important to prevent deleterious ribosomal frameshift mutations. However, comparison of *mnmE* and *gidA* (*mnmG*) mutant phenotypes indicates that GidA has additional functions outside tRNA modificiation (Bregeon et al., 2001). The other well-conserved gene flanking the replication origin is *mioC*, located immediately rightward of *oriC* (Figure 1). *mioC* encodes a protein that has been implicated in biotin synthesis *in vitro* (Birch et al., 2000), but *mioC* mutants do not require biotin for growth in rich or minimal medium (D.B., unpublished). Thus, MioC protein has no established biological function.

Transcription of both *mioC* and *gidA* are thought to contribute to regulation of replication initiation, as *oriC* plasmids require both genes for replication (Lobner-Olesen et al., 1987; Asai et al., 1990; Bates et al., 1997). Since open reading frame deletions within either gene are generally not deleterious (Tanaka and Hiraga, 1985), it is thought that transcription through these genes affects replication initiation by changing origin topology (Asai et al., 1990). Based on the twin-domain supercoiling model (Liu and Wang, 1987), *gidA* transcription oriented away from *oriC* would introduced stimulatory negative supercoils, and *mioC* transcription oriented toward *oriC* would introduce inhibitory positive supercoils (Figure 1). This so-called “transcriptional activation” model is supported by the fact that *gidA* and *mioC* transcription is strongly cell-cycle specific, with stimulatory *gidA* transcription highest before initiation and *mioC* inhibitory transcription highest after initiation (Theisen et al., 1993; Su'etsugu et al., 2003). However, these effects seem to be specific to *oriC* plasmids. Preventing transcription from either gene on the chromosome has no measurable phenotype on the timing, rate or synchrony of replication initiation in otherwise wild-type cells under a variety of growth conditions (Lobner-Olesen and Boye, 1992; Bates et al., 1997; Asai et al., 1998; Molina et al., 1999). Only when multiple DnaA binding sites were deleted within *oriC*, was *gidA* transcription found to stimulate chromosomal initiation (Bates et al., 1997). These studies and the high sequence conservation of both *mioC* and *gidA* suggest that transcription near *oriC* may have once been an important initiation mechanism but, as we will argue here, may have later evolved other roles.

In a previous study, we observed that *mioC* and *gidA* promoter mutations in a *fis*^−^ cell background resulted in extreme cell filamentation (Bates et al., 1997). Fis is a well-conserved and abundant nucleoid associated protein that is important for chromosome structure and transcriptional regulation of many genes, but has no known role in cell division (Skoko et al., 2006; Bradley et al., 2007; Browning et al., 2010). The expression of *fis* is stringent controlled and Fis levels rise dramatically after nutritional upshift reaching a maximum in mid-exponential phase (Mallik et al., 2006). Fis binds within the *oriC* sequence and likely has some role in replication initiation (Filutowicz et al., 1992) particularly under rapid growth conditions (Flatten and Skarstad, 2013). Because DNA/mass values (an indicator of replication efficiency) of *fis* mutant cells were not further reduced by *mioC* and *gidA* promoter mutations (Bates et al., 1997), we concluded that cell filamentation in the triple mutant resulted from some unknown feature of cell division regulation. Here, we further examine how these three genes affect DNA replication, chromosome segregation and cell division.

## Materials and methods

### Bacterial strains, plasmids and growth conditions

All strains used in this work are derivatives of *E. coli* MG1655. Genotype, construction and sources of all strains are provided in Table S1. Unmarked gene deletions in *sulA* (DB768) and *fis* (DB670) were created by amplifying a kanamycin resistance gene cassette flanked by Flp recognition sequences (FRT) from pKD4 (Cherepanov and Wackernagel, 1995), with 40 bp fragments on both ends homologous to the 5′ and 3′ ends of *sulA* or *fis* (primers listed in Table S2), then transferring the construct into the MG1655 chromosome by homologous recombination (Yu et al., 2000) selecting for kanamycin resistance. The *kan* gene was then deleted by expression of Flp from pCP20 (Cherepanov and Wackernagel, 1995) and curing the plasmid. The *ymgF::tet* deletion mutation was created by amplifying the tetracycline resistance gene from pBR322 with 40 bp fragments on both ends homologous to the 5′ and 3′ ends of *ymgF* (primers listed in Table S2), then transferring the construct into MG1655 by homologous recombination. A strain with in-frame *gidA* and *mioC* deletions was made by transferring a linearized *gidA122*Δ *mioC*Δ*121 asnA101::cat* fragment from the mutant *oriC* plasmid pDB123 (Bates et al., 1997) into MG1655 by homologous recombination. The *ymgF::tet* and *PsulA-GFP* mutations were introduced into various *PgidA PmioC fis* strains by P1 transduction. Wild-type *oriC* plasmid pDB101 is previously described (Bates et al., 1997). All strains were verified by PCR or sequencing.

For all experiments, cells were grown in LB medium supplemented with 0.1% glucose at 37°C. Unless otherwise stated, exponential growth was achieved by 1:1600 dilution of a saturated culture into fresh medium with shaking to OD600 absorbance of 0.2, at which point samples were removed for analysis. Antibiotics were added for selection in P1 transduction and to maintain plasmids at the following concentrations: ampicillin (50 μg/ml), chloramphenicol (50 μg/ml), tetracycline (20 μg/L) and kanamycin (55 μg/ml).

### Flow cytometry and qPCR

DNA content was measured in DAPI stained cells by flow cytometry as previously described (Joshi et al., 2013). For rifampicin runoff analysis, exponential cultures were treated with 150 μg/ml rifampicin to inhibit replication initiation and 10 μg/ml cephalexin to inhibit cell division, and incubated at 37°C with shaking for 1 h to allow completion of replication. All flow cytometry was performed on a Becton-Dickinson LSR II Cell Analyzer. *oriC* to *ter* ratios were determined by quantitative reat-time PCR (qPCR) using the ΔΔC_*t*_ method. Primers and details for qPCR are previously described (Joshi et al., 2011).

### Phase and fluorescence microscopy (cell length, PI, DAPI, FM4-64, FISH)

For cell length determination, cells were fixed in 2.5% formaldehyde for 15 min at room temperature then 45 min on ice, followed by three washes in PBS pH7. Cell monolayers were created by applying fixed cells between a coverslip and an agarose-coated slide, then imaged by phase contrast. Cell length was determined in 500–600 cells per sample using MicrobeTracker software (Sliusarenko et al., 2011). For membrane permeability assays, live cells were washed three times in 0.85% NaCl and stained with 5 μM propridium iodide and imaged by fluorescence microscopy. The fraction of fluorescent (PI-positive) cells were quantified for 1000–1500 cells per sample by hand counting. Nucleoids and cell membranes were visualized in formaldehyde-fixed cell samples by simultaneous staining with 0.5 μg/ml 4′, 6′-diamidino-2-phenlyindole (DAPI) or 1 μg/ml FM4-64. FISH analysis of *oriC* and *ter* positioning was performed as previously described (Joshi et al., 2011).

### Transcriptome analysis

RNA for RNA-seq was isolated using acid phenol extraction and sequencing libraries were prepared using the Bacteria ScriptSeq kit (Epicentre). Briefly, rRNA was depleted from 5 μg total RNA using the Ribo-Zero Bacterial rRNA removal kit (Epicentre), followed by isopropanol precipitation of mRNA. Resulting mRNA was converted to terminal-tagged cDNA and 15 cycles of PCR were performed using ScriptSeq Set 1 index PCR primers (Illumina) to create the indexed cDNA libraries. These were quantified by Qubit fluorimetry and qPCR and diluted to a final concentration of 18 pM and pooled. Single-end sequencing was performed on an Illumina Next-generation MiSeq sequencer using a 150-cycle MiSeq Reagent Kit V3. Reads were aligned using Rockhopper software (McClure et al., 2013), using MG1655 as a reference genome. Transcript levels of *PmioC* (DB535) and *PgidA* (DB679) samples were normalized to a wild-type (DB510) sample. Statistical analysis and graphing were performed in Matlab (MathWorks).

## Results

### Blocking *mioC* and *gidA* expression inhibits cell division

**Figure 2:**
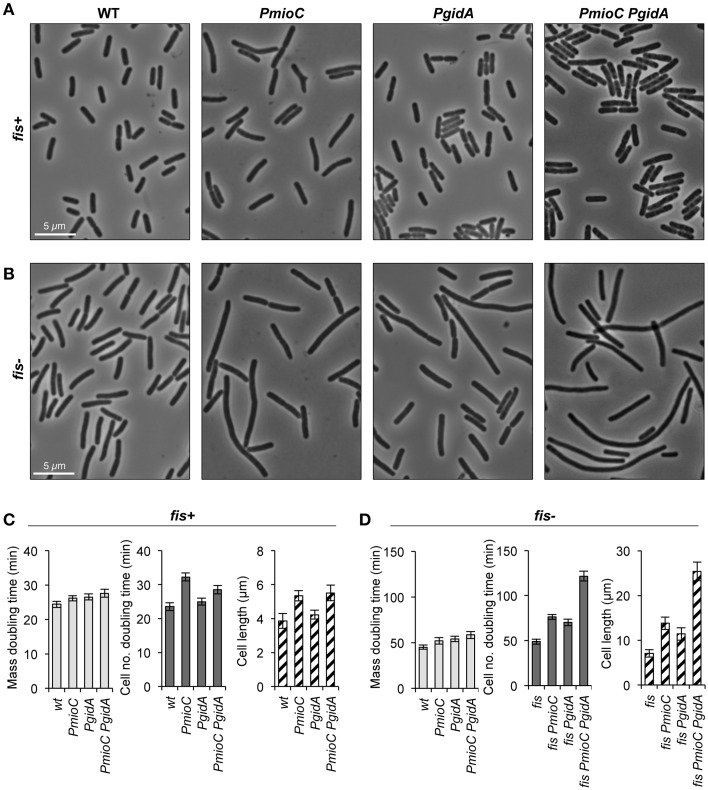
***mioC*** **and** ***gidA*** **promoter mutants are elongated**. Cells carrying promoter mutations (*PmioC, PgidA*) in a *fis*^+^or *fis*^−^ genetic background were diluted 1:1600 in LB medium at 37°C and assayed for growth and cell division. **(A,B)** Phase contrast images of cells at mid-exponential phase (OD600 = 0.2). **(C,D)** Above cultures were assayed for mass doubling time by optical density at 600 nm (light gray bars), cell number doubling time by flow counting in a Coulter particle counter (dark gray bars), and cell length at OD600 = 0.2 by direct microscopic observation (hatched bars, *n* = 500–600 cells). Values are means of 5 independent experiments ±1 standard deviation (SD).

In a previous study demonstrating that *mioC* and *gidA* transcription do not affect chromosomal replication initiation, we created promoter mutations in each of the genes (*PmioC112* and *PgidA103*) that resulted in severely decreased transcription as implied previously from ß-galactosidase assays (Bates et al., 1997) and verified here by RNA sequencing (Figure 1, red). Although *PmioC* and *PgidA* mutations did not affect the rate or timing of replication initiation (Bates et al., 1997), they did result in subtle increases in cell length, especially *PmioC* which was ~35% longer than isogenic wild-type (Figures 2A,C; hatched columns). Continued cell growth in the absence of cell division was reflected in a disparity between mass (OD600) doubling time and cell number doubling time measured using a particle counter (Figure 2C; light and dark gray columns).

The addition of a *fis::kan* null mutation greatly exacerbates the cell elongation phenotype of the promoter mutations, with triple mutant *fis PmioC PgidA* exhibiting severe filamentation (Figures 2B,D; hatched columns). Although *fis*^−^ cells have a reduced growth rate (~50% of wt; Figures 2C,D), neither *PmioC* nor *PgidA* significantly enhanced the poor growth phenotype of the *fis* mutant (Bates et al., 1997; below) suggesting that the promoter mutations affected cell division directly and not through general growth effects. Similar cell elongation was observed in MG1655 (used here) and CM735 (not shown) strain backgrounds. Interestingly, despite a severe cell division phenotype in triple mutant cells, the strain is viable with fewer than 1% of cells stainable with propidium iodide (data not shown). Survival is explained by the ability of filamentous cells to divide in late exponential and early stationary phase, with stationary phase cultures consisting of ~normal length cells (Figure S1). Fis mediates expression of a diverse set of genes involved in all aspects of growth including translation, nutrient transport, carbon and energy metabolism, but relatively few genes involved in DNA replication and cell division (Bradley et al., 2007).

### Cell division inhibition is independent of both the SOS response and nucleoid occlusion

**Figure 3:**
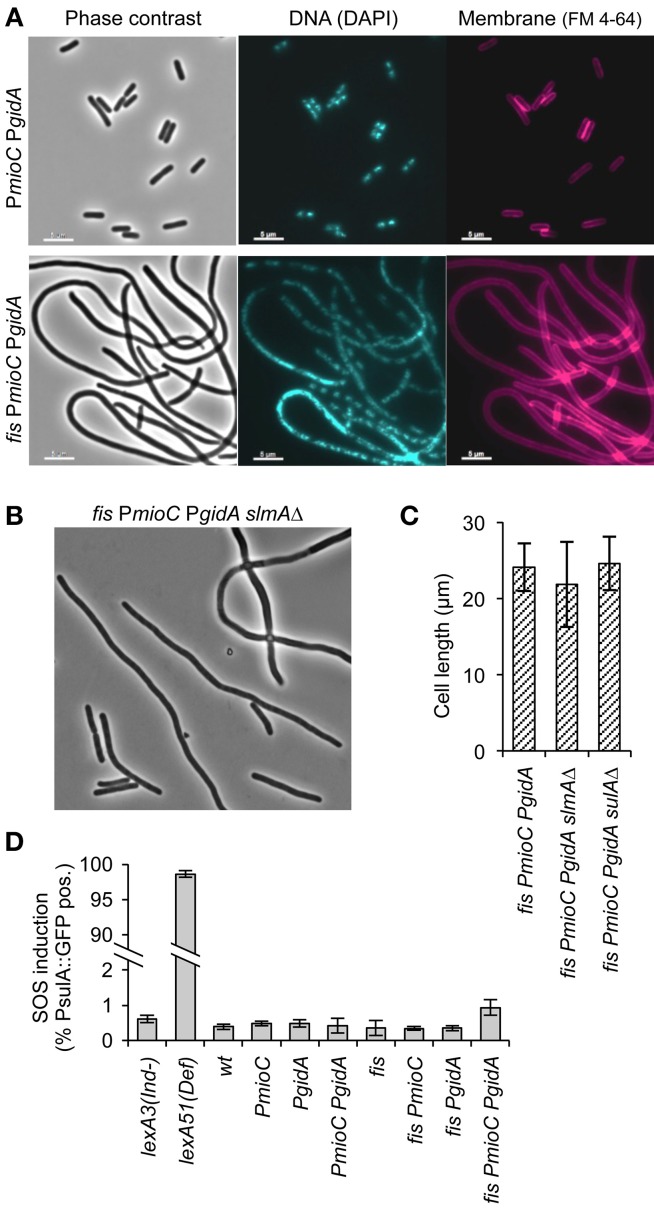
**Promoter mutants do not activate the SOS response or nucleoid occlusion. (A)** DNA and membrane staining in a *PmioC PgidA* double mutant (top) or *PmioC PgidA fis* triple mutant (bottom) indicate an absence of division septa between segregated nucleoids. **(B)** Phase contrast image of a *PmioC PgidA fis slmA*Δ quad mutant. **(C)** Cell length of triple mutant (*PmioC PgidA fis*) cells in the presence or absence of a *slmA* or *sulA* deletion mutation. **(D)** SOS induction in *PmioC PgidA* and *fis* mutant combinations was measured by flow cytometry of strains carrying a *PsulA::GFP* reporter construct (Materials and Methods). Values are means of 3 independent experiments (±1 SD).

An early stage of cell division is inhibited in triple mutant cells as indicated by the absence of division septa in filamented cells (Figure 3A, right). Further, we observed very few anucleate cells, which would occur frequently if FtsZ rings were repositioned by nucleoid occlusion in cells with unsegregated DNA (Bernhardt and De Boer, 2005). Supporting the idea that division was not inhibited by a chromosome segregation failure, nucleoid staining with DAPI showed that most filaments contained well-separated nucleoid bodies presumably composed of fully replicated chromosomes (Figure 3A, middle). Rare triple mutant cells exhibiting diffuse DAPI staining also frequently stained positive for propidium iodide, indicating that non-segregated nucleoids in those cells may have resulted from cell lysis. We further tested whether cell elongation in triple mutant cells was independent of nucleoid occlusion by introducing a *slmA*Δ mutation, which prevents the inhibition of midcell FtsZ rings over unsegregated DNA (Bernhardt and De Boer, 2005). We observed no significant decrease in cell length of quad mutant *slmA*Δ *fis PmioC PgidA* over *slmA*^+^ *fis PmioC PgidA* cells (Figures 3B,C), nor was there an increase in frequency of anucleate cells (not shown). This suggests that chromosome segregation was not significantly perturbed as the filamentous phenotype was not caused by nucleoid occlusion of the division plane.

We next asked whether cell elongation was dependent on the SOS response, which activates a large normally repressed regulon of cell cycle arrest and DNA repair genes that includes the potent cell division inhibitor, SulA (Huisman et al., 1984). To test if SOS was activated in elongating cells, we measured expression of a *PsulA*-GFP reporter gene inserted into the chromosome of all mutant strains (Shee et al., 2013). Cells were grown exponentially and GFP fluorescence was measured by flow cytometry. All mutant strains, including the filamentous triple mutant exhibited <1% of green cells during exponential growth (Figure 3D). Subsequent analysis of *PsulA*-GFP expression in triple mutant cells by microscopy, indicated that even among the longest cells (>15 μm), <5% were SOS-induced (not shown). In contrast, constitutively SOS induced *lexA51(Def)* cells were ~98% GFP-positive, validating the assay. We further introduced a *sulA* deletion into the triple mutant, and found no difference in cell length compared to triple mutant *sulA*^+^ cells (Figure 3C). We conclude that cell elongation observed in our mutants was independent of both the two cell division inhibitors SulA and SlmA.

### Cell elongation did not result from inefficient chromosome replication or segregation

**Figure 4:**
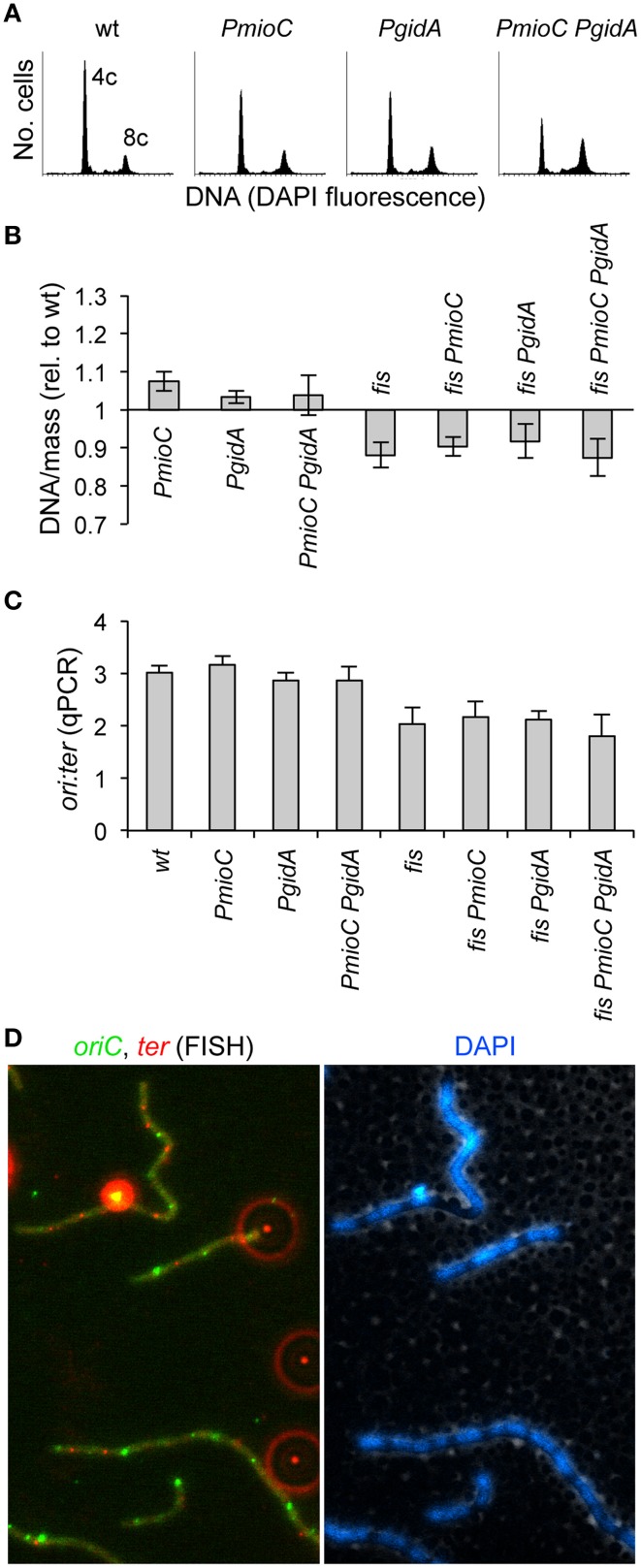
**Promoter mutations do not impair DNA replication or chromosome segregation. (A)** Rifampicin runoff DNA histograms in *PmioC* and *PgidA* mutants indicate synchronous replication initiations. Numbers represent the number of chromosomes at the major peaks. **(B)** DNA/mass in exponentially growing cultures was measured by flow cytometry of DAPI stained cells (values are relative to *wildtype*). **(C)** The ratio of *oriC* sites to *ter* sites in above cells was measured by qPCR, indicating the rate of DNA replication and whether replication forks were blocked between *oriC* and *ter*. Values in **B,C** are means of 3 independent experiments (±1 SD). **(D)** The positions of *oriC* (green foci) and *ter* (red foci) in elongating triple mutant *PmioC PgidA fis* cells was analyzed by FISH (left panel). Bright extracellular foci (appearing as red halos) are multi-fluorescent beads added for image alignment. DAPI staining (right panel) indicates *ter* foci are located between segregated nucleoids, consistent with normal chromosome segregation (Text). See Material and Methods for details.

We previously reported that promoter mutations in *mioC* and *gidA* did not adversely affect the timing or efficiency of chromosomal replication (Bates et al., 1997). Even a subtle delay in replication could create an unbalanced cell cycle that results in delayed cell division. For example, blocking replication elongation by depleting dNTPs with hydroxyurea results in incomplete replication intermediates that block division independently of both SOS and SlmA (Cambridge et al., 2014). To confirm and extend our previous analysis of DNA replication, we measured DNA content and cell mass by flow cytometry in the mutant set. As expected, promoter single and double mutants in a *fis*^+^ background exhibited a normal number of chromosomes and synchronous replication initiation, as shown by rifampicin runoff histograms (Figure 4A). In this method, which involves blocking replication initiation with rifampicin and cell division with cephalexin and allowing ongoing replication forks to complete, cells accumulate a number of fully replicated chromosomes equal to the number of origins present at the time of drug treatment. Under our growth conditions (LB/37°C), wild-type cells mostly contained either 4 or 8 origins (Figure 4A, left). This indicates that replication initiation occurred synchronously, with all origins in a cell firing ~simultaneously, and thus containing 2^n^ origins where n is equal to any positive integer. Double *PmioC PgidA* mutants exhibited slightly more 8-origin cells than *wildtype*, but this difference was not statistically significant. Rifampicin runoff in *fis*^−^ cells did not produce discernable chromosome peaks in our flow cytometry analysis, likely owing to asynchronous replication (Flatten and Skarstad, 2013) and uneven DAPI staining due to abnormal chromosome structure (Skoko et al., 2006).

The ratio of DNA per unit of cell mass in exponentially growing cells (without rifampicin treatment), is essentially constant in healthy populations, but differs strongly in strains with inefficient replication initiation or elongation (Stepankiw et al., 2009 and references therein). DNA/mass was unaffected by *mioC* or *gidA* promoter mutations in either *fis*^+^ or *fis*^−^ backgrounds as determined by DAPI and light scatter flow cytometry measurements (Figure 4B), implying that cell elongation did not result from a reduced replication initiation or elongation rate, which would decrease DNA/mass values. By comparison, temperature-sensitive *dnaA46* mutant cells grown at 30°C, which are about twice the length of identically-grown wild-type cells, exhibit a 30–40% reduced DNA/mass value (Boye et al., 1996).

We further looked for evidence of slowed replication elongation by measuring the *oriC* to *ter* rato in our mutants by quantitative PCR (qPCR). This assay would reveal for example any replication fork blockage late in the C period, which would likely not significantly affect overall DNA/mass values. Wild-type cells had an *ori*:*ter* ratio of ~3, which was unchanged in single or double promoter mutants (Figure 4C). Similarly the *ori*:*ter* ratio in *fis*^−^ cells (≈2) did not significantly change upon introduction of either or both *mioC* or *gidA* promoter mutations. To test whether chromosome segregation was impaired in our mutant, which could account for inhibited cell division (above), we examined the number and positions of *oriC* and *ter* copies by fluorescence *in situ* hybridization (FISH). This analysis showed evenly spaced *oriC* and *ter* foci in all strains, even in elongated triple mutant cells (Figure 4D). These data suggest that cell elongation present in triple mutant cells did not result from a reduction in the rate of replication initiation or blocked replication elongation or chromosome segregation.

### The division block is mediated by a lack of MioC and GidA protein

**Figure 5:**
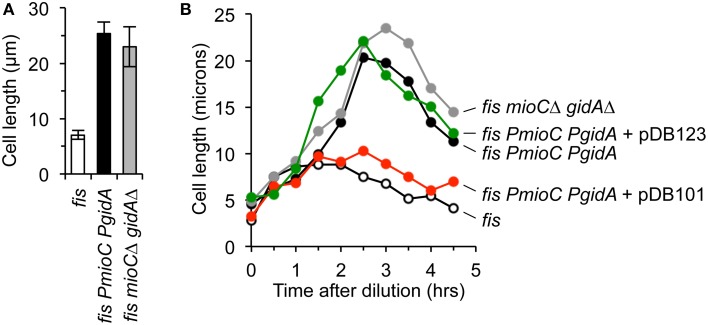
**The division phenotype of** ***mioC*** **and** ***gidA*** **mutants is complemented** ***in trans***. **(A)** Cell length in exponential cultures of *fis* parental, *fis* with *mioC* and *gidA* promoter mutations, and *fis* with *mioC* and *gidA* in-frame ORF deletions with active transcription, was measured by direct microscope observation (3 independent experiments, ±1 SD). **(B)** Cell length was measured over time through all three growth phases in the indicated mutants and plasmids. Complementation of the cell division phenotype in triple mutant cells (*fis PmioC PgidA*) occurred with a plasmid expressing MioC and GidA protein (pDB101), but not with a control plasmid carrying *mioC* and *gidA* ORF deletions (pDB123).

The transcriptional activation model (Theisen et al., 1993) posits that *mioC* and *gidA* primarily function to modulate replication initiation via transcription-induced supercoiling changes at *oriC*. Given that all our tests indicate that *PmioC* and *PgidA* mutants have unperturbed chromosomal replication initiation, fork elongation, and chromosome segregation, it is a reasonable prediction that cell elongation in promoter mutants is due to an absence of MioC and GidA proteins, and not gene transcription *per se*. To test this idea, we created in-frame deletion mutants of both genes, in which most of the open reading frame was deleted but promoters were left in-tact (Materials and Methods). Transcription through the remaining open reading frame occurs at normal levels and an *oriC* plasmid containing both mutations could support plasmid replication (Bates et al., 1997). Cells carrying a *fis* mutation with both *mioC*Δ and *gidA*Δ in-frame deletions exhibited severe cell elongation (Figure 5A, gray), with average cell length within error of *fis PmioC PgidA* cells (Figure 5A, black). Examination of cell length through all three growth phases showed that both the *fis*^+^ double mutant and the *fis*^−^ triple mutant exhibited similar temporal patterns of elongation with maximal cell length occurring in late exponential phase (Figure 5B, gray and black lines). These results support the idea that *mioC* and *gidA* affect cell division by providing a division-related gene product, not by transcription-induced replication effects.

We next asked whether the cell division phenotype of triple mutant cells could be complemented *in trans* by exogenous GidA and MioC protein. Cells carrying *PmioC PgidA* and *fis*^−^ mutations were transformed with two *oriC* plasmids, pDB101 carrying the wild-type *mioC* and *gidA* genes and promoter sequences, and pDB123 carrying the *mioC*Δ and *gidA*Δ in-frame deletions. Both of these plasmids fully support *oriC*-dependent plasmid replication (Bates et al., 1997). Triple mutant *fis PmioC PgidA* cells expressing GidA and MioC protein from pDB101 grew to a maximum length of ~7–10 microns, similar to cells carrying a single *fis* mutation (Figure 5B, red and open circles). By comparison, *fis PmioC PgidA* cells transformed with the control plasmid that does not express MioC or GidA protein but has a functioning *oriC* (pDB123), elongated to a maximum of ~22 μm (Figure 5B, green) with similar extent and kinetics as non-transformed triple mutants. We conclude that MioC and GidA are cell division regulatory proteins.

### MioC and GidA regulate expression of a cell division gene, *ymgF*

**Figure 6:**
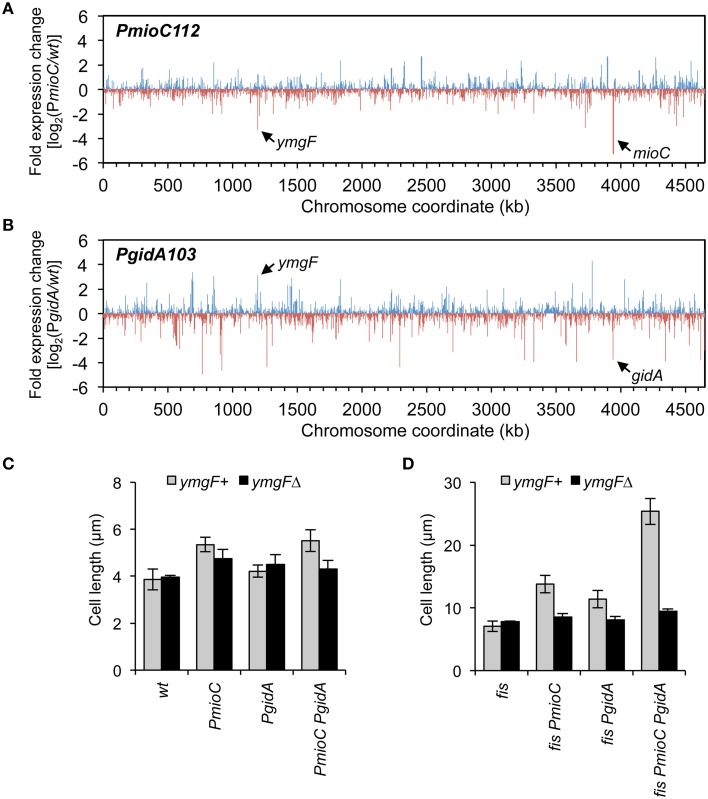
**Transcription profiling in** ***mioC*** **and** ***gidA*** **mutants. (A,B)** Changes in gene expression across the *E. coli* genome in *mioC* **(A)** and *gidA* **(B)** promoter mutants relative to *wildtype* was determined by RNA sequencing (Materials and Methods). Blue and red ticks indicated positive or negative fold-changes of individual genes. The positions of *mioC* and *gidA*, and a strongly affected cell division gene, *ymgF*, are indicated. See Table S3 for description of other affected genes. **(C,D)** The cell elongation phenotype of *gidA* and *mioC* mutants requires YmgF. Cell length was measured in exponentially growing *wildtype* and *mioC* and *gidA* promoter mutants in *fis*^+^ **(C)** or *fis*^−^ **(D)** genetic background, in the presence (black bars) or absence (gray bars) of a *ymgF*Δ mutation.

Although neither MioC nor GidA are known to regulate transcription, both have been implicated in information transfer functions (Introduction) and it is possible that mutants have altered expression of one or more cell division-related genes. To test this, we measured the relative abundance of all the *E. coli* mRNAs in wild-type and *PmioC* and *PgidA* single mutant strains by next-generation RNA sequencing. Both mutants had several up-regulated and several down-regulated genes as shown by high and low ticks, with each tick representing a single gene (Figures 6A,B). Altered genes included various ontology groups including amino acid and carbon metabolism, transcription, membrane proteins, and small molecule transport (Table S3). One gene in particular, *ymgF*, was significantly under-expressed (3.3-fold less than *wt*) in the *PmioC* mutant and over-expressed (3.1-fold more than *wt*) in the *PgidA* mutant (Figures 6A,B, arrows). This gene was previously identified in a bacterial two-hybrid screen for factors that interact with the division septum component FtsL (Karimova et al., 2009). GFP-tagged YmgF localizes strongly to the division septum, and although a *ymgF* deletion does not confer a growth or cell division phenotype, overexpression of YmgF suppresses a temperature-sensitive *ftsQ* mutant (Karimova et al., 2009). YmgF is a 72 amino acid integral membrane protein, but little else is known about its function.

To test epistasis relationships between *mioC, gidA*, and *ymgF*, we examined cell length in our mutant set in the presence or absence of a *ymgF::kan* deletion mutation. Cell elongation in the *PmioC PgidA* double mutant was decreased by ~20% after addition of *ymgF*Δ, with resulting cell lengths within error of wild-type cells (Figure 6C). This effect was greatly amplified in *fis*^−^ derivatives, with nearly a nearly 3-fold reduction in cell length of quad mutant cells (*fis PmioC PgidA ymgF*Δ) compared to isogenic *ymgF*^+^ cells, to approximately wild-type lengths (Figure 6D). These results suggest that the entirety of MioC's and GidA's effect on cell division is dependent on YmgF, and therefore they act upstream of YmgF to regulate division.

## Discussion

### MioC and GidA promote cell division without affecting replication initiation

We have shown that *mioC* and *gidA* mutants have a reduced capacity to divide, which is exacerbated by a *fis* deletion. Several data suggest that division inhibition did not result from late or inefficient DNA replication. (1) Mutations in *mioC* and *gidA* did not reduce the simultaneous firing of multiple origins in the same cell (initiation synchrony). (2) DNA contents (DNA/mass or number of origins) were not affected by a *mioC* or *gidA* mutation, even in the presence of a *fis* mutation. (3) Division inhibition did not involve SOS induction, which could have resulted from DNA damage caused by replication defects, as shown by an absence of PsulA-GFP expression in triple mutant *PmioC PgidA fis* cells and by normal *sulA* expression in *PmioC* and *PgidA* strains by transcription profiling. (4) Elongating *mioC* and *gidA* mutants showed no apparent chromosome segregation defects (e.g., that might result from late DNA replication) by DAPI analysis and by FISH analysis of *oriC* and *ter* numbers and locations. (5) Triple mutant cells continued to elongate in the presence of a *slmA* mutation, suggesting that division was not inhibited by nucleoid occlusion. (6) Finally, the cell division defect was shown to result from a lack of MioC and GidA protein, not a lack of *mioC* and *gidA* transcription, which has previously been implicated in replication initiation control (Introduction).

All of these results suggest that *mioC* and *gidA* affect cell division independently of any *cis*-mediated effects at *oriC*, and this is supported by work indicating that *mioC* and *gidA* do not affect chromosomal replication initiation (Lobner-Olesen and Boye, 1992; Bogan and Helmstetter, 1996; Bates et al., 1997). Possible subtle segregation defects capable of inhibiting division but undetected by DAPI or FISH analysis cannot be ruled out. Additionally, it was recently shown that unsegregated chromosomes can block cell division independently of SOS or SlmA (Cambridge et al., 2014), leaving open this possibility. Transcription analysis in *Salmonella gidA* mutants, which also exhibit cell elongation, indicated abnormal expression of several chromosome segregation genes including *mreB, mukB, xerC, parA*, and *parB* (Shippy et al., 2012). In our transcription analysis, none of these genes, nor any other known segregation gene, were significantly changed in either *PgidA* or *PmioC* strains, thus there may be a divergence of roles for GidA and MioC between the two organisms.

### Expression of *mioC* and *gidA* are cell cycle controlled

**Figure 7:**
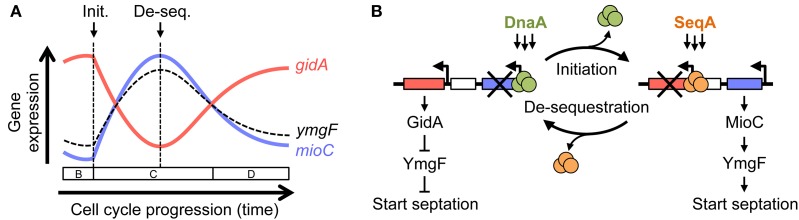
**A model for MioC and GidA-mediated linkage between replication initiation and cell division. (A)** Schematic of *mioC* (blue) and *gidA* (red) transcription during the cell cycle from synchronized cell experiments (Theisen et al., 1993; Ogawa and Okazaki, 1994), and predicted expression of *ymgF* (dashed curve). Times of replication initiation and origin de-sequestration are indicated by arrows, with cell cycle pre-initiation (B period), replication (C period), and cell division (D period) shown above time line. **(B)** Cyclic inverse expression of *mioC* and *gidA* occur as a consequence of replication-dependent remodeling and subsequent remethylation (de-sequestration). See Discussion for detailed description.

Transcription of many genes varies significantly during the cell cycle (Zhou et al., 1997), which in many cases is correlated in time and amplitude to the predicted replication of those genes, but in others, like *mioC* and *gidA*, transcription is switch-like with abrupt on/off kinetics (Theisen et al., 1993; Ogawa and Okazaki, 1994; illustrated in Figure 7A). Both *mioC* and *gidA* transcription is thought to be mediated by the initiation proteins DnaA and SeqA, whose binding sites overlap the *mioC* and *gidA* promoters (Figure 1A). Binding of these proteins is primarily regulated by remodeling events that occur at *oriC* at the time of initiation (Figure 7B): (1) A cluster of GATC sites in the left end of *oriC* become hemimethylated after passage of the replication fork, which causes ~20-fold increase in SeqA binding immediately adjacent to the *gidA* promoter (Slater et al., 1995; Nievera et al., 2006). Transcription of *gidA* is continually blocked until *oriC* is remethylated ~1/3 of the cell cycle later (the sequestration period; Slater et al., 1995; Bogan and Helmstetter, 1997). (2) At the same time, *mioC* transcription is de-repressed by replication initiation, through removal of DnaA protein bound to two conserved DnaA boxes within the *mioC* promoter (Bogan and Helmstetter, 1996). DnaA rebinding is thought to be temporarily inhibited by titration of free DnaA to newly replicated binding sites, which are concentrated toward *oriC* on the *E. coli* chromosome (Kitagawa et al., 1998). (3) Remethylation of the GATC cluster in *oriC* and subsequent removal of SeqA protein (de-sequestration) de-repress the *gidA* promoter. (4) Finally, the cycle is completed by rebinding of the *mioC* promoter by DnaA protein, blocking its transcription. This cyclic expression of GidA and MioC protein allows us to develop a working model for a new replication-division linkage regulatory pathway (below).

### General model for replication initiation-dependent cell division control

The resulting *mioC* and *gidA* transcription pattern places maximal MioC expression about midway through DNA replication at which point GidA expression is at its lowest (Figure 7A). From our expression analysis, transcription of *ymgF*, encoding a division septum protein (Karimova et al., 2009), was ~3-fold higher in *gidA* mutants and ~3-fold lower in *mioC* mutants.

Joining these data, we predict that *ymgF* expression would reach a maximum about midway through DNA replication, or just after, very near the point at which FtsZ polymerization normally begins (e.g., Addinall and Lutkenhaus, 1996). During fast growth, replication initiation at multiple origins is synchronous, thus maintaining cyclic and coordinated *mioC, gidA, and ymgF* expression. Modulation of *ymgF* expression by MioC and GidA may involve a complex indirect mechanism, as neither protein are known transcription regulators (Introduction).

The weak division phenotype of *mioC* and *gidA* mutants (without an additional *fis* mutation) as well as a *ymgF*Δ mutant (Karimova et al., 2009), suggests that the affected division control mechanism is likely either redundant with other control mechanisms, or part of a division checkpoint that is utilized under specific growth phases, or both. Because Fis is a known regulator of replication initiation (Filutowicz et al., 1992; Flatten and Skarstad, 2013) with no known role in regulating division, we speculate that deletion of *fis* exacerbates the division phenotype of *gidA* and *mioC* mutants by delaying replication initiation. Late *oriC* firing (and *gidA* and *mioC* promoter remodeling) would normally lead to a corresponding late division cycle with adjusted (late) division, but there is no such adjustment in the *gidA mioC fis* triple mutant, thus severe elongation. Further research into if and how MioC, GidA, and YmgF interact to mediate cell division, as well as how Fis contributes to this mediation is essential.

The above model is consistent with the proposal by Nordstrom and colleagues that DNA replication and cell division are independently regulated processes that are temporally correlated through the actions of specific “cell cycle” checkpoints (Nordstrom et al., 1991; Boye and Nordstrom, 2003). This idea stemmed from observations that changes in the timing of DNA replication initiation brought about by manipulations of growth medium or temperature were not accompanied by closely corresponding changes in the timing of cell division (i.e., cell length; Bernander and Nordstrom, 1990; Boye et al., 1996).

### Two-trick ponies?

Considering the substantial published data on *mioC* and *gidA* (not half of which is cited here) we speculate that these well-conserved genes have dual function, mediating cell division in response to replication initiation, and also activating *oriC* under exceptional circumstances. We previously showed that strains with a large deletion within the minimal *oriC* sequence required *gidA* transcription for viability (Bates et al., 1997), implying that *gidA* transcription stimulates open complex formation in these cells. Similarly, these genes may help drive initiation under conditions of low DnaA, temperature, or other suboptimal initiation conditions, and they may have been an essential component of a primordial initiation system (Asai et al., 1998). Transcription of *mioC* and *gidA* may also regulate initiation (and division) in non-steady state situations, in which the normal 1:1 ratio of initiation and division is temporarily circumvented to support multi-forked replication (in rich media there are division-less initiations in lag phase and initiation-less divisions in early stationary phase). This idea is supported by the fact that *mioC* is stringently controlled (Chiaramello and Zyskind, 1989). Interestingly, *mioC* transcription is affected by a putative cell division protein, MraZ, which binds a site overlapping the the *mioC* promoter DnaA box (Eraso et al., 2014). Cells overexpressing MraZ have ~12-fold reduced *mioC* transcription, and exhibit severe cell elongation to the point of cell death, but MioC overexpression did not alleviate the cell division defect (Eraso et al., 2014), therefore the relationship between MraZ-mediated division inhibition and *mioC* repression is unclear. Additionally, *mioC* and *gidA* genes may be responsible for over-initiation of replication observed after thymine depletion (Martín et al., 2014). Thymine starvation results in stalled replication forks, and cells typically die from an abundance of stalled forks near *oriC*. Martín and colleagues showed that thymine-less death could be prevented by introducing promoter mutations in *mioC* and *gidA*, suggesting that these genes are part of a (sometimes futile) cell cycle response pathway to reinitiate replication when fork progression is impaired.

### Conflict of interest statement

The authors declare that the research was conducted in the absence of any commercial or financial relationships that could be construed as a potential conflict of interest.

## Supplementary material

Figure S1 **Growth phase-specific cell elongation in** ***PmioC PgidA fis***. Cell length (open circles) and culture density (OD600, dashed line) were measured in *PmioC PgidA fis* triple mutant cells for 10 h after 1:1600 dilution into fresh LB medium. Maximal cell length occurs in mid-late exponential phase, with cells approaching normal cell length by late stationary phase.

Table S1 ***E. coli* strains**.

Table S2 **Cloning primers**.

Table S3 **Functional categorization of GidA and MioC regulated genes^*^**.
