# Supplementary figures and images for: MioC and GidA proteins promote cell division in E. coli
Source: Front Microbiol. 2015 May 28;6:516. doi: 10.3389/fmicb.2015.00516 (PMC4446571; doi:10.3389/fmicb.2015.00516)

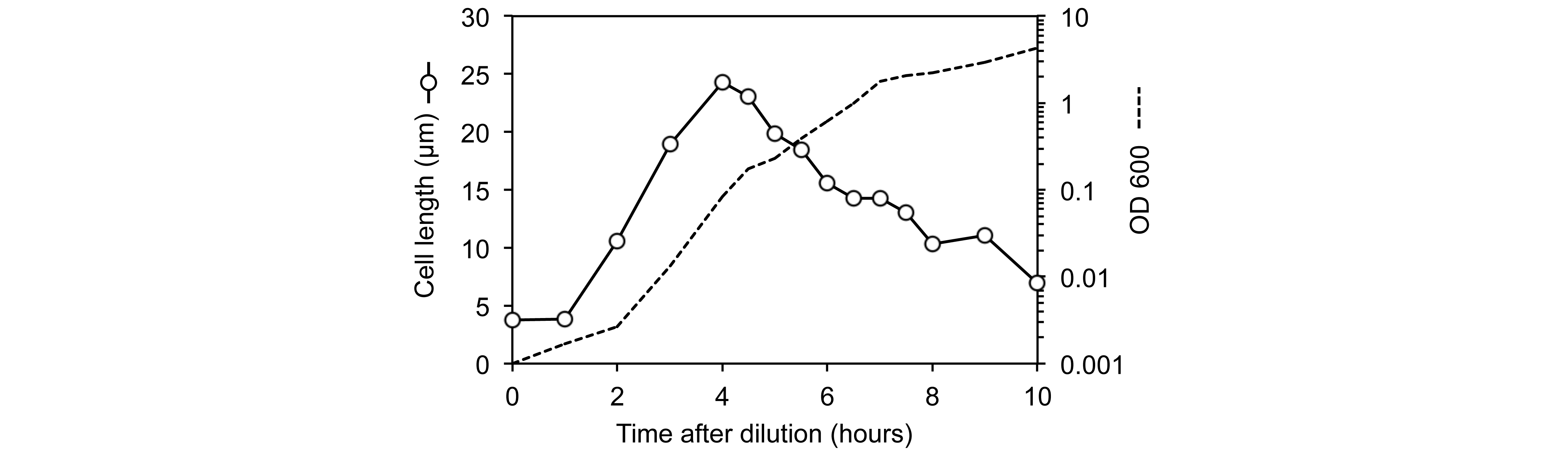

Supplement: Figure S1 — Growth phase-specific cell elongation in PmioC PgidA fis. Cell length (open circles) and culture density (OD600, dashed line) were measured in PmioC PgidA fis triple mutant cells for 10 h after 1:1600 dilution into fresh LB medium. Maximal cell length occurs in mid-late exponential phase, with cells approaching normal cell length by late stationary phase. [file Image1.TIF]
