# Supplementary material for: MioC and GidA proteins promote cell division in E. coli
Source: Front Microbiol. 2015 May 28;6:516. doi: 10.3389/fmicb.2015.00516 (PMC4446571; doi:10.3389/fmicb.2015.00516)
Supplement: Table S1 — E. coli strains. [file TableS1.DOCX]

| **Strain** | **Genotype** | **Construction and Source** |
| --- | --- | --- |
| DB510 | *asnA101::cat* (as *wildtype*) | MG1655 x P1.AQ9648 (Bates et al., 1997) |
| DB535 | *asnA101::cat PmioC112* | MG1655 x P1.AQ10293 (Bates et al., 1997) |
| DB679 | *asnA101::cat Pgid103* | MG1655 x P1.AQ9652 (Bates et al., 1997) |
| DB537 | *asnA101::cat PmioC112 Pgid103* | MG1655 x P1.AQ10614 (Bates et al., 1997) |
| DB543 | *asnA101::cat fis::kan* | DB510 x P1.WM2016 (Koch et al., 1988) |
| DB539 | *asnA101::cat PmioC112 fis::kan* | DB535 x P1.WM2016 (Koch et al., 1988) |
| DB700 | *asnA101::cat Pgid103 fis::kan* | DB679 x P1.WM2016 (Koch et al., 1988) |
| DB571 | *asnA101::cat PmioC112 Pgid103 fis::kan* | DB537 x P1.WM2016 (Koch et al., 1988) |
| DB768 | *sulA∆* (complete ORF deletion) | MG1655 linear recombination (see Methods) |
| DB779 | *sulA∆ fis::kan* | DB768 x P1.WM2016 (Koch et al., 1988) |
| DB802 | *sulA∆ fis::kan asnA101::cat PmioC112 Pgid103* | DB779 x P1.AQ10614 (Bates et al., 1997) |
| DB670 | *fis∆* (complete ORF deletion) | MG1655 linear recombination (see Methods) |
| DB1726 | *fis∆ asnA101::cat PmioC112 Pgid103* | DB670 x P1.537 |
| DB1802 | *fis∆ asnA101::cat PmioC112 Pgid103 slmA::kan* | DB1726 x P1.JW5641 (Baba et al., 2006) |
| DB1876 | *asnA101::cat mioC121∆ gidA122∆* | MG1655 linear recombination (see Methods) |
| DB1877 | *asnA101::cat mioC121∆ gidA122∆ fis::kan* | DB1876 x P1.WM2016 (Koch et al., 1988) |
| DB2597 | *ymgF::tet* | MG1655 linear recombination (see Methods) |
| SMR6039 | *att::ΩPsulA-GFP* | (Hastings et al., 2004) |
| SMR868 | *lexA3*(Ind-) | (Galhardo et al., 2009) |
| SMR5400 | *lexA51*(Def) *sulA∆* | (Galhardo et al., 2009) |

**Table S1. *E. coli* strains**

**Strain references**

Baba, T., Ara, T., Hasegawa, M., Takai, Y., Okumura, Y., Baba, M., Datsenko, K.A., Tomita, M., Wanner, B.L., and Mori, H. (2006). Construction of *Escherichia coli* K-12 in-frame, single-gene knockout mutants: the Keio collection. *Mol Syst Biol* 2**,** 2006 0008.

Bates, D.B., Boye, E., Asai, T., and Kogoma, T. (1997). The absence of effect of *gid* or *mioC* transcription on the initiation of chromosomal replication in *Escherichia coli*. *Proc Natl Acad Sci U S A* 94**,** 12497-12502.

Galhardo, R.S., Do, R., Yamada, M., Friedberg, E.C., Hastings, P.J., Nohmi, T., and Rosenberg, S.M. (2009). DinB upregulation is the sole role of the SOS response in stress-induced mutagenesis in Escherichia coli. *Genetics* 182**,** 55-68. doi: 10.1534/genetics.109.100735.

Hastings, P.J., Slack, A., Petrosino, J.F., and Rosenberg, S.M. (2004). Adaptive Amplification and Point Mutation Are Independent Mechanisms: Evidence for Various Stress Inducible Mutation Mechanisms. *PLoS Biology* 2**,** 2220-2233.

Koch, C., Vandekerckhove, J., and Kahmann, R. (1988). *Escherichia coli* host factor for site-specific DNA inversion: Cloning and characterization of the *fis* gene. *Proceedings of the National Academy of Sciences USA* 85**,** 4237-4241.
