# Supplementary material for: MioC and GidA proteins promote cell division in E. coli
Source: Front Microbiol. 2015 May 28;6:516. doi: 10.3389/fmicb.2015.00516 (PMC4446571; doi:10.3389/fmicb.2015.00516)
Supplement: Table S2 — Cloning primers. [file TableS2.DOCX]

| **Primer** | **Sequence** |
| --- | --- |
| *sulA∆* Forward | CTGTACATCCATACAGTAACTCACAGGGGCTGGATTGATTtgtgtaggctggagctgcttcg |
| *sulA∆* Reverse | TGGGCGACAAAAAAAGTTCCAGGATTAATCCTAAATTTACgtacatatgaatatcctccttagttcct |
| *fis∆* Forward | AAAATTTTGCGTAAACAGAAATAAAGAGCTGACAGAACTgtgtaggctggagctgcttcg |
| *fis∆* Reverse | GAGTAGCGCCTTTTTAATCAAGCATTTAGCTAACCTGAATTATTCGAacatatgaatatcctccttagttcct |
| *ymgF::tet* Forward | TTTGTTTGCTATAATTGTTTGAAAGTTTTGACAGGATTGCCATTAGTAGCAATTCTCATGTTTGACAGCTTA |
| *ymgF::tet* Reverse | ACTGCCAGCAACCCATTCAGCCGCATACTGACAGTGAGAAACTGAAAGATTCAGGTCGAGGTGGCCCGGCTC |

**Table S2. Cloning primers**
