# Supplementary material for: MioC and GidA proteins promote cell division in E. coli
Source: Front Microbiol. 2015 May 28;6:516. doi: 10.3389/fmicb.2015.00516 (PMC4446571; doi:10.3389/fmicb.2015.00516)
Supplement: Table S3 — Functional categorization of GidA and MioC regulated genes*. [file TableS3.DOCX]

**Table S3.**  **Functional categorization of GidA and MioC regulated genes*.**

| **Functional category** | **GidA-regulated genes**  **(fold change in mutant)** | **MioC-regulated genes**  **(fold change in mutant)** |
| --- | --- | --- |
| Amino acid biosynthesis, metabolic process and respiration | *grcA* (-3.1); *ddpX* (+2); *dmsA* (-3.3); *narG* (-4.4); *napA* (-2.8); *napD* (-4.3); *napF* (-4.4); *sdhD* (+2.6); *sdhC* (+3.3); *sdhA* (+2.1); *sgcQ* (-2.3); *thiE* (-2); *yhbV* (-3.0); *yhcC* (-4); *ynjE* (-2.1); *yjjY* (-3); *yjjI* (-3.8); *ynfK* (-2.4); *tdcA* (-2.1); | *trpE* (-2); *tdcC* (+2.3); *tdcB* (+2.3); *tnaA* (+2.6); *tnaB* (+2.6); *tnaC* (+2.7) |
| Transcription | *glnK* (-2); *feaR* (+2.3); *glcC* (+2.1); *hcaR* (*+*2.1); *lldR* (+2.7); *mhpR* (+2.5); *ttdR* (-2); *ydcI* (+2.8); *yjhI* (-2.6) | *pyrL* (-2.3); *rybB* (-2) |
| Membrane protein, small molecule transport | *cusF* (-2); *dcuC* (-2.9); *entB* (-2.5); *entC* (-2.6); *entE* (-2.6); *fepG* (-2.2); *focA* (-3.3); *feoA* (-2.4); *lldP* (+3.4); *aldA* (+2.3); *nikA* (-4); *nikC* (-3.3); *ompW* (-2.6); *potG* (+2); *ugpB* (2.5) | *glpC* (+2.3); *yicS* (+2); *blc* (-2); *dsdX* (+2.6); *malG* (+2.6); *nanC* (+2.3); *yhjX* (-3.2) |
| Cell division | ***ymgF*** **(+3.1)** | ***ymgF* (-3.3)** |
| DNA replication, DNA repair | *mmuM* (-2.3) | *umuD* (-2.1) |
| Unknown function | *ydjY* (-2); *yegJ* (-2); *yjdK* (-3.9); *yjfI* (-2); *yigI* (+2.8); *yodD* (-2.3) | *yjfY* (-3); *yjfJ* (-2) |
|  |  |  |

* Expression levels were determined by RNAseq. Only genes expressed > 2-fold differently than WT are shown (fold change indicated in parentheses).
